# Experiences of Recovery in EPAPSY’s Community Residential Facilities and the Five CHIME Concepts: A Qualitative Inquiry

**DOI:** 10.3389/fpsyt.2020.00024

**Published:** 2020-02-13

**Authors:** Antigoni Apostolopoulou, Stelios Stylianidis, Philia Issari, Panagiotis Chondros, Amalia Alexiadou, Pepy Belekou, Charalambos Giannou, Eleni K. Karali, Vana Foi, Fotini Tzaferou

**Affiliations:** ^1^ Department of Psychology, National and Kapodistrian University of Athens, Athens, Greece; ^2^ Department of Psychology, Panteion University for Social and Political Sciences, Athens, Greece; ^3^ Association for Regional Development and Mental Health (EPAPSY), Athens, Greece

**Keywords:** recovery, CHIME model, community residential care, qualitative inquiry, thematic analysis

## Abstract

In the context of the reform of psychiatric services in Greece, the debate about the concept of recovery is still growing. Recovery is defined as a path through which individuals facing mental health challenges are enabled to regain and further develop significant relationships with family, friends, the community, and themselves and at the same time to cope with the detrimental effects of stigma through empowerment. The present qualitative study aims to explore the experiences of people living in EPAPSY’s (Association for Regional Development and Mental Health) community residential facilities focusing mainly upon the key concepts of the CHIME (connectivity, hope, identity, meaning, and empowerment) conceptual framework of recovery. To this end, semi-structured interviews were conducted with seven participants living and receiving mental health care in a residential facility of EPAPSY. The participants’ accounts were analyzed using thematic analysis in a deductive and inductive manner. The research findings highlighted, among others, the challenges the participants faced during their early years in family and school, the experience of the revolving door effect, and the perceived turn their life took when they were transferred to a community residential facility, thus opening a new chapter in their lives. Of the five CHIME concepts, all are present in the participants’ accounts, with emphasis given to a meaningful present, a need to feel “normal” again, and a positive outlook for the future, both for themselves and their relationships, despite the persistence of certain difficulties.

## Introduction

In the context of the reform of psychiatric services in Greece, the debate about the concept of recovery is growing, as in many other European countries (1–3). Core values of the recovery model, as they will be presented below, but also the WHO and European Union (EU) directives and guidelines for the organization of mental health services (4–7), all highlight the importance of developing and implementing policies and strategies that promote the active engagement and participation of users of services and their family in the mental health system. As Amering [(8): vii] suggests:

*“recovery demands all our best efforts in terms of human rights, patients’ rights, scientiﬁc and clinical responsibility and service, in the interest of those of us who might become patients and those who have. We learn from those who are using services, those who have used services (ex-users) and those who deﬁne themselves through overcoming harmful experiences in the support system (survivors).”*

Recovery is often seen as a philosophy, a critical way to approach mental health. Describing the conceptual framework of recovery has been difficult and for this reason, research on issues of implementation strategies and techniques, effectiveness, and limitations has started to grow only during the last decade (9, 10). For Spaniol and Koehler (11), recovery is defined as a path through which individuals facing mental health challenges are enabled to regain and further develop significant relationships with family, friends, the community, and themselves and at the same time to cope with the detrimental effects of stigma through empowerment. Necessary aspect of the recovery process is refinding and reconstructing a new meaning in life for the person struggling with mental health challenges (12). Based on the definition proposed by Deegan (13), recovery refers to the real living experience of the people receiving mental health care. Leamy et al. (14) have suggested the CHIME (connectedness, hope, identity, meaning, and empowerment) model of recovery as an explanatory framework. CHIME is the acronym for connectedness; hope and optimism about the future; identity; meaning in life; and empowerment, as basic components comprising recovery.

Each of these five concepts comprise of a number of different but interrelated notions (15). Starting with connectedness, the concept includes the support provided by peers and others, the user’s active participation in the community, as well as the development of meaningful relationships with family, friends, and other individuals. Hope comprises of a positive outlook for the future ahead, a deep-seated belief in the possibility of recovery, finding and keeping motivation for change, sustainment of hope-inspiring relationships, an overall optimism for life and personal development, as well as having dreams and aspirations for life. Identity deals with agency and personal responsibility, the perceived capability to escape from something undesirable and overcoming stigma, and an overall re-creation of the self. Meaning comprises of finding goals and social roles in life, constructing a personal understanding of mental illness, finding spirituality, and leading a life with quality. Finally, empowerment is described as the development of personal agency and control over personal choices, with particular attention to each person’s strengths and the ability to regain control over personal health care (15).

In Greece, research on recovery is extremely limited. On the contrary, in Europe and the United States, the number of reviews is growing on various aspects: conceptual approach (14, 15), implementation strategies (16–19), measuring, process and outcome evaluation (10, 20, 21), and the role of professionals and users.

Leamy et al. (14) undertook a review study in order to gather, compare, and organize the available literature on models of recovery. A modified thematic synthesis based on the papers of this review identified 13 characteristics of the recovery journey that form the CHIME processes. A short version of INSPIRED tool (17) is discussed that can cover the CHIME dimensions (22). The Questionnaire about Process of Recovery (QPR) (23, 24) is considered to contribute to the investigation of the CHIME framework. A literature review by Shanks et al. (25) aimed at identifying measurable key factors of recovery. Their findings highlighted the QPR (24) as the most efficient in measuring recovery under the CHIME framework. Other studies investigated the extent to which services and professionals can follow the CHIME framework (17, 26, 27). It has also been used as a foundation for a new measure of recovery support from services (28).

Brijnath (29) studied the possible cultural effects on the CHIME model, focusing on two culturally diverse groups in Australia and their members’ experience with recovery from depression. Using qualitative interviews and thematic analysis, she found that participants were faced with discriminatory behavior and attitudes by family members on the basis of their mental health difficulties; having a positive attitude toward their recovery from depression helped them maintain an optimistic outlook for the future; ambivalence toward medication, which was perceived both as a major help toward recovery and as a constant reminder that they will never get back to their healthy self; the indigenous group found meaning through spirituality, whereas the Anglo-Australian group constructed meaning through the experience of depression itself; and that for both groups, the notion of agency and empowerment was translated into a sense of social and economic security [(29) 664]. CHIME was applicable in both groups, but there were cultural differences in the ways that participants in each group perceive some of the key notions of the model.

Piat et al. (30) used observation and interviews in an effort to outline the way that CHIME (14) is reflected on everyday life and to highlight the contribution of the model’s key notions to the recovery of individuals with mental health challenges. Their findings suggest that the CHIME model (14) can better inform our understanding of the recovery process.

Very recently, Piat et al. (31) inquired into the role that choice plays in the recovery process for persons with mental health problems living in supported residential facilities. Using qualitative interviews, they concluded that living in a residential facility strengthens tenants’ ability to take personal responsibility and make choices concerning their everyday activities and routine, like cooking and shopping groceries. This, in turn, underlined their sense of regaining a “normal” life, in which their views and preferences are voiced and respected by the other tenants, staff members, and professionals. Richter and Hoffman (32) have also looked into the concept of choice but regarding the initial choice on living settings rather than everyday issues during supported living. In other words, they reviewed and meta- analyzed parts of published studies focusing on service users’ preference of independent housing facilities.

Williams et al. (33) focused on the service users’ perceptions of the CHIME model notions of connectedness and hope using visual methods. Participants were asked to watch a video concerning the lived experience of psychosis and to talk about their feelings about it in semi-structured interviews. Findings showed that the experience of watching the video was quite powerful for the participants, strengthening their sense of hope and connectedness. In other words, participants felt that they are not alone out there struggling with mental health difficulties and that there is a hopeful future ahead of them, as there is for other people with the same mental health problems.

Therefore, within this framework, the present study aimed to help fill the gap in the study of recovery in the Greek context attempting to explore the experiences of individuals accessing mental health care in a community residential facility. More specifically, it adopted a qualitative approach in order to map out the experiences of people living in one of EPAPSY’s (Association for Regional Development and Mental Health) community residential facilities, focusing on participants’ understanding of personal recovery and pertinent themes related to the key concepts of the CHIME (connectedness, hope, identity, meaning, and empowerment) (14) conceptual framework of recovery.

## Methods

**Figure 1:**
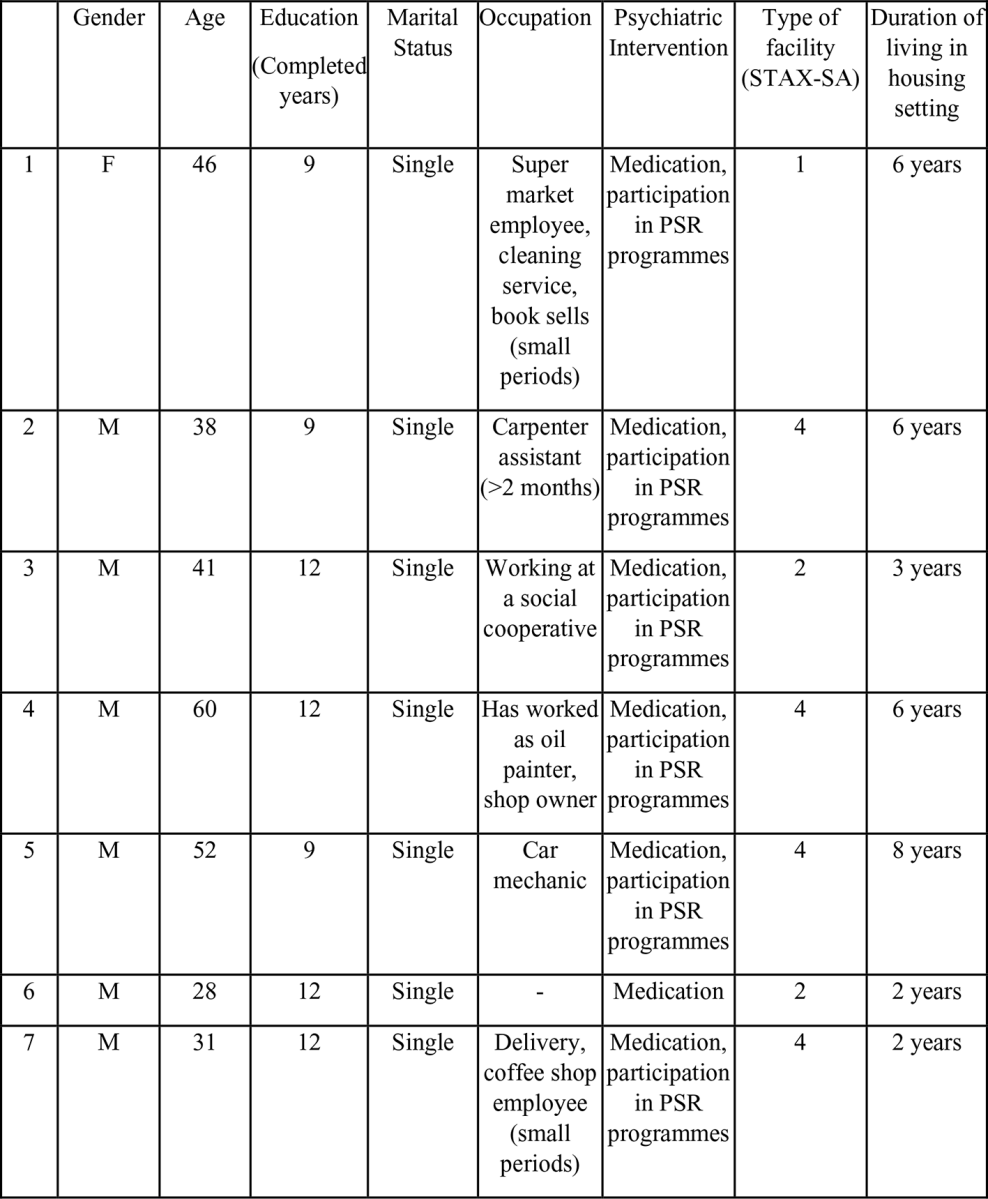
Participants in the study. F, female; M, male; PSR, Psychosocial rehabilitation.

In order to adequately address the aims of the study, qualitative methodology was adopted as it offers the opportunity for the voices of individuals accessing mental health care to be heard and for in-depth exploration of their personal understanding of recovery (34, 35). Moreover, the study was informed by the theoretical framework of recovery-CHIME (14). The research was conducted at the psychosocial rehabilitation units of the Association for Regional Development and Mental Health—EPAPSY—a nongovernmental, nonprofit organization which was founded in 1988. EPAPSY is operating a total of 25 residential facilities. There is a variation between them regarding the number of residents, number of staff, if there is staff on-site, and criteria for receiving accommodation support. Out of these 25 units, 12 (protected accommodation apartments) have four residential places, there is no staff on-site, they offer low/moderate support, and there is limited emphasis on move-on and support individual accommodation [type 4 according STAX-SA, (36)]. Eight have 15 places, staff on-site, high support, limited emphasis on moving on a congregate setting (type 1). Five have 10 places, staff on-site, strong emphasis on move-on, high support, congregate setting (type 2). The number of staff varies between 13 and 27 people (psychologist, social worker, nurse, carer, psychiatrist, clinical supervisor, and administrator). One of these facilities is for adolescents (between 12 and 18 years old). **Figure 1** shows participants in the present study, main descriptive demographics, and the facility type from which they were recruited.

### Sample

Participants in the present study were seven individuals (one woman and six men, 28–60 years of age), who agreed to share their experiences and understanding of recovery. They all reported previous experiences of two to four hospitalizations in various psychiatric institutions across Greece. Of the seven participants, one is female aged 46 and living in a high-support facility with limited emphasis on moving-on (type 1 in STAX-SA taxonomy) for 6 years. Of the six remaining men, all aged between 28 and 60, four live in a protected apartment for 2–8 years, and two live for the past 2–3 years in high-support settings but with strong emphasis on moving-on (type 2 in STAX-SA taxonomy). Only one is working full time, and one is a pensioner. No one is married. They all take part in team activities, foreign language lessons, training courses, and creative workshops. Participants were recruited based on convenience and purposeful sampling, taking into account their availability, ability to participate with their consent to the interview process, as well as the number of years living in the community residential facility (minimum 2 years).

### Data Collection

Collection of data was made using semi-structured interviews. Interview questions were open-ended and focused upon participants’ experiences before the onset of the mental illness, their personal understanding of recovery, their present life in the residential facilities, and aspirations for the future. Interviews were conducted by members of the therapeutic team, thus providing a safe and familiar setting for participants. Acknowledging the risk of biased answers from the participants, due to the fact that their recollections and experiences where described to a member of the therapeutic personnel, it should be noted that it was considered the optimal option in order to create a caring environment that would not disrupt the participants’ lives and relations. Prior to the interviews, the researchers informed the service users of the aims of the study and gave details of the confidentiality of their personal information. Interviews were recorded and transcribed verbatim so as to capture in detail participants’ experiences. The interview questions centered around prior experiences with mental health challenges, hospitalizations, present life in residential facility, personal life and relationships, and plans for the future.

### Data Analysis

The research material which resulted from the interviews was analyzed and categorized into main themes and subthemes in accordance with the principles of thematic analysis and the six steps proposed by Braun and Clarke (37, 38), namely, familiarization with the data, initial coding, initial emergence of themes, refinement of themes, definition and naming of themes, and reporting of findings.

In the present study, thematic analysis was conducted both inductively and deductively (39) informed by the conceptual framework of CHIME. Specifically, thematic analysis was first conducted inductively to explore the main themes emerging from participants’ accounts, holding no prior theoretical or other expectations [e.g., (38, 40)]. Then, drawing on the CHIME conceptual framework, researchers worked deductively in order to explore the presence of connectedness, hope, identity, meaning, and empowerment in participants’ experiences and personal understanding of recovery. Working deductively and adopting an “interpretation of suspicion” (41), researchers are better able to convincingly describe and situate the phenomenon under study based on a certain theoretical and/or conceptual model that defines and explains the key concepts explored (34, 35, 42).

## Results

The research findings reflect the experiences of people living in EPAPSY’s residential facilities and more specifically the CHIME key notions relating to recovery. From the analysis of participants’ narratives emerged three main themes and a number of subthemes related to the time before the onset of mental illness, the period of hospitalization, and the present life of service users living in the community residential facilities (**Figure 1**). Among other things, participants’ accounts reflected notions related to the CHIME recovery framework, such as connectedness, hope, meaning, identity, and empowerment.

### Before the Onset of Mental Illness

Service users described the period in their lives before the onset of mental illness and the beginning of hospitalizations. Their narratives focused upon family, school, behavior-related issues, and their socioeconomic situation.

#### Family

All participants seemed to have experienced family difficulties during their childhood. As one of the service users pointed out:

*“…from the beginning of their relationship my parents had problems, due to my mother’s mental health issues and the fact that my father was Turk and my grandfather and grandmother could not accept him. When I was 3 years old my father left us. So did my mother, when I was 6 years old. My grandmother took custody of me, but due to her health issues [dementia] she couldn’t take care of me …”* [H.T.]

Another participant recalled:

*“My parents never married; I barely remember my father—he left when I was very young. I always felt my mother distanced.”* [CH.R.]

#### School

Participants also described multiple challenges experienced during their school years such as difficulties related to school attendance and the need for work, learning problems, and strained relationships with their schoolmates.

*“I went to primary school and then high school … I couldn’t continue school because I had to work as a cheesemaker, because my father got sick and he couldn’t work. But I was already working, I was working since I was 4 years old.”* [E.K.]

*“I never went to kindergarten. In primary school, which I finished with difficulty, I had serious learning problems, and I didn’t have any friends, because often the other kids were making fun of me.”* [H.T.]

#### Behavior-Related Issues

As they grew up and moved through adolescence and early adulthood, a number of behavior-related issues emerged including addictive behavior, violence, and delinquent behaviors:

Addictive Behavior

One participant recalled that

*“… [along with my brother] we would gamble, horseraces … all the money our father gave us. I was out of line.*” [M.F.]

Still another described his life before the onset of mental illness and hospitalizations as a period where he only worked, used substances, and had phobias.

Violence and Delinquent Behavior

For some service users, violence and delinquent behavior were also present in their lives. As M.F. pointed out:

“*I had a girlfriend and I beat her; I was jealous of her and I beat her … I wanted her to cry…*”

“*We would deceit old people … we were out of line, it is fraud, you can go to jail for it. We would deceit old people and get their money.*”

#### Poverty and Multiple Losses

At the same time, participants’ socioeconomic situation was in dire straits, with poverty and multiple losses being the dominant elements in their lives.

Poverty

As S.M. narrated,

“… *at that time, I quit my job, there was unemployment, and little by little, there came poverty*.”

Another participant described her life in the streets:

“… *I remember living in the streets … it rained and it snowed and I had to sleep on the benches. I had a coat and I wore it and I slept alone and people would give me some change in order to get something to eat and there were “one-night lovers” who were bothering me. And the police would take me in for identification* …” [M.L.]

*Multiple Losses*

Along with poverty, multiple losses were present in participants’ lives:

“… *After a series of misunderstandings my relationship came to an end, I had a row with my best friend, I got fired, I had to leave my house … I had no money to live by and I sold all my furniture, I had lost all my friends and my relationship with my family was really bad. I decided to live in the car, which was parked outside my mother’s house. But that didn’t last for long, due to economic hardship I also sold the car*” [CH.R.]

In all, service users drew a rather bleak picture of their past which was dominated by poverty, various losses, loneliness, delinquent/abusive behavior with outbursts and substance or/and alcohol abuse, as well as difficulties in finding and keeping jobs, social relations, and housing.

### Hospitalization

#### Revolving Door

Participants’ accounts of the period in their lives after the onset of mental illness and hospitalization stressed the circularity of the pathway chronic patients follow within the psychiatric care system, which begins with an admission to a psychiatric clinic, adhering to a therapeutic plan, exiting the clinic, relapsing, readmitting, etc., a pattern which has been described as the Revolving Door effect (43, 44).

E.K. described the revolving door effect in his own words:

“… *In 2004, as I was quarrelling again with my neighbors, I threw a rock at the window of one’s house. So, they proceeded with the public prosecutor and I was hospitalized … Then a long time passed, during which I was hospitalized for a few months, I returned home, got into problems with the neighbors, the legal process with the prosecutor was repeated, and I would get hospitalized again* …”

This “drifting” (45) of patients in the psychiatric treatment system is associated with negative consequences for their lives, that is, reduced chances of rehabilitation, weakened belief in their ability to improve their lives and health, exacerbated unemployment, and of course economic exhaustion of themselves and their families (46).

#### Emotions

Another important aspect related to the period of hospitalizations was the presence of strong and negative emotions such as fear, anxiety, suffering, loneliness, suspicion, tension, and frustration.

### Present Life in a Community Residential Facility

Participants reported experiences and narrated stories related to finding new meaning in their lives, positive emotions, a sense of connectedness and support networks, occupational rehabilitation, a movement toward a positive self-image, a sense of empowerment, and hope for the future associated with their residence in community residential facilities such as a boarding house, a hostel, or sheltered apartments. The service users talked about the quality in their present lives, a willingness to be alive, the acquisition of new friends, receiving support from the therapists, and having recovered their relationship with their family and relatives. Additionally, they emphasized education and work as central in their struggle to regain life and identity. As opposed to the bleak image of their past, they communicated a sense of security and normality and described the warmth they feel at present. Moreover, they talked of their dreams for the future, and they stressed the sense of independence and freedom they experience as they gain more autonomy in their lives.

#### Connectivity

The service users reported changes in their lives as soon as they were transferred to community residential facilities and narrated experiences that come close to notions of connectivity as proposed by the CHIME recovery framework. These included supports given by people with similar problems, help from friends acquired in the context of the residential facility, renewed relationships with family, social networks, and a sense of belonging to a community (15).

*Support From Others—Being Part of a Community*

In the words of Z.E.:

“*I have support, I have solidarity, I have people who are interested … I feel that I can become a member of society, I am a part of society … I have learned how to approach people, I’ve learned myself … I want to be more sociable*…”

In a similar manner, M.L. commented:

“*Here, people helped me to get myself together.*”

Along these lines, E.D. added:

“*I would say that anyone who feels ‘over-stretched,’ as I did, shouldn’t fight with everyone and everything, as I did, but should ask for help*…”

Another important point was made by CH.R.:

“*Within a year, my everyday life became creative, and to that effect it was the friends that I made both inside and outside the hostel that helped.*”

“*Socially, I have friends, I feel more peaceful.*” [S.M.]

“*I love my friend N. [new friend, from the community]*” [M.F.]

In all, support from others and a sense of belonging to a community helped service users to regain a renewed interest in life, to turn to new activities, and to develop their creativity.

*Renewed Relationships With Family*

At the same time, participants described renewed relationships with their parents and siblings.

“…*I now see my parents under a new light; our relationship was always strained. It was hard for me to acknowledge my brother as part of the family. I thought my parents were my enemies. I love and respect myself and so I feel for my family members. I believe that I can show them that. I have already seen them a few times. Our meetings aren’t as they used to be*…” [Z.E.].

“*Now I go for a vacation every year for one month at my sister’s house at the village where I was born*.”

#### Hope

Participant’s accounts regarding their present lives reflected a positive attitude, which stands in stark contrast to their difficult past. They portrayed notions of hope as proposed by the CHIME recovery framework referring to optimism and dreams for the future, the belief in the possibility of recovery, the existence of an incentive for change, hope-inspiring relationships, and positive thinking (15, 47). At the same time, there was awareness of obstacles in fulfilling hopes and dreams.

Positive Attitude

In the words of Z.E.:

“*I want to find a girl, to make many trips, to dream … to have a family, to have a normal job and to earn more than I now earn, to have friends, to live in Athens, to visit and to be visited by my family regularly, to have humor, not to have difficulty expressing my feelings, to have a dog, to study, to buy a house … to have less difficulty in communication.*”

While maintaining a positive attitude toward the future, services users seemed also to be aware of obstacles and constraints to their hopes and dreams.

*Awareness of Difficulties in Fulfilling Hopes and Dreams*

While maintaining a positive attitude, M.F. was also aware of certain difficulties that he has to deal with:

“*Whenever the eye symptom catches me, thoughts begin in my mind but now it catches me less. When it catches me, I try to manage it as well as possible*…”

Likewise, CH.R. stated:

“*I see that there are difficulties in dealing with many things. I know I want a lot … I will never be able to do it all by myself, still it is the end result that counts.*”

Along these lines, even though M.L. was able to express her hopes and dreams for her future, she situated their fulfillment in the distant future:

“*For the future, I wish I were a housewife with my husband, in our little home, going for a walk on Sundays, both having a job, and having our home open for our friends and sleeping without voices … to be friends with other couples … But this dream is far away*…”

#### Identity

Service users’ narratives reflected also the process of redefining the self-image through a positive light and overcoming the stigma of mental illness (14). Although the notion of identity seems to overlap with other concepts of recovery as also noted by Stuart et al. (15), renegotiation of a positive image for the self seems to be an integral part of the recovery process—and of identity change. In our study participants, assumption of responsibilities, the feeling that they were active members of society, a sense of agency in their social and family relations, reclaiming normality, and occupational rehabilitation seemed to result in regaining control of their lives, which in turn strengthened their sense of identity and self-worth (15, 48).

In the words of CH.R.:

“… *I have more responsibilities but more freedom. I feel more secure and it’s like I live again on my own, I now have my own keys for the house. It is an important step in my life … Things are getting better for me; I feel my life is on a pleasant track.*”

For CH.R., acquiring his own keys for his home seemed to act as a confirmation of his existence as an autonomous and independent member of society which gave him a sense of joy. It is this sense of freedom and personal agency that also paves the way for a redefinition of personal identity (49).

The process of consolidating a positive self-image is also reflected in the words of Z.E.:

“*I now love and respect myself, and my family members. I think I can show it to them.*”

For S.M., a good self-image is associated with the notion of normality:

“*I feel like a normal person again … I feel good for all new things happening in my life … I feel I have a normal life* …”

S.F. talked about a positive sense of identity acquired through occupational rehabilitation:

“*In 2014, I opened the shop … a gift shop. I have decorated the space on my own, it has frames, jewelry, decorative items, everything. I began a new chapter, I felt really good. Immediately the problems disappeared and I felt good. It was a good experience for me. I felt like a normal person, I had no mental health problems, there was nothing stressing me … During the summer of 2017, there was another chapter, the social cooperative X. I got elected as vice president … I believe everything will turn up right* …”

Along these lines, E.K. reported that he was involved in a social cooperative, a place as he said “*where there may be a job opening for me to work at …*”

Similarly, CH.R. stressed the importance of acquiring a job:

“*I want to work again, I talked to some people … to find a job. I found a subsidized bakery–pastry seminar for three months, which I successfully completed. Things got better for me and I feel that my life is turning good for me and has meaning*.”

Almost all participants talked about the value of work which seemed to strengthen their sense of independence, agency, and personal responsibility, making them feel as active and important members of the community and contributing to meaning making.

#### Meaning Making

Within the CHIME framework, the concept of meaning refers to the process by which people regain a meaningful life, understand mental illness and the difficulties surrounding it, turn to spirituality in order to form a framework of understanding and explanation of their lives and experiences, seek an active role in society, and work toward their well-being (15). In our study, the service users emphasized the value of meaning-making activities and experiences. After their transition to a community residential facility, participants got involved in a series of activities which were meaningful to them and promoted personal well-being and quality in their lives. Moreover, they contributed to a sense of responsibility and agency that helped them develop a new awareness of their possibilities and strengths as well as an active role in the community.

“*We are doing fine … We have our daily routine, we come to the boarding house for our medicines, we go to the bakery, to the butcher’s, to the supermarket, we go out for coffee. I’m responsible for the daily cooking, I write down everything we will need from the supermarket, and the other two are doing the cleaning*.” [E.K.]

“…*I find pleasure in various activities, like vacation, visits to other places and team membership. At the same time, I haven’t stopped ‘hunting’ for a job*.” [CH.R.]

“*I have warmth, I have my bed, my food, my activities. I feel like I have a family now … I am grateful … Thanks to the activities I get to know people, I learn something and my mind gets smarter. I can speak a little English, since I’m attending English lessons. I can play a part in a theater play with a false face … it means pretending to be somebody else. Comedy, that is. Comedy helps me, because drama doesn’t help me that much. Drama makes you cry, whereas it’s better to laugh*.” [M.L.]

“*I have quality in my life, I like living and that’s a bit new to me … I’m happy for every new day dawning; I didn’t feel that in the past … I feel that I can become a member of society, part of society*.” [Z.E.]

We are reminded of Yalom (50) words related to meaning seeking, engagement, and existence:

“*To find a home, to care about other individuals, about ideas or projects, to search, to create, to build—these, and all other forms of engagement, are twice rewarding: they are intrinsically enriching, and they alleviate the dysphoria that stems from being bombarded with the unassembled brute data in existence*” (p. 482).

#### Empowerment

Empowerment refers to the process by which the individual regains control of his or her life, assumes personal responsibility for himself/herself, and invests in the positive elements of his/her personality. Empowerment emphasizes that it is not enough to have a supportive network, but that each individual needs to actively seek to regain and change his or her life (15).

As reflected in M.F.’s account:

“… *in the apartment, I was more comfortable, more constructive … I wanted to do things to feel better. I went to the gym … in the past, gym was not for me, I was not going. Did it help? Yes, very much*.”

Z.E. also talked about exercise, among others things:

“… *I learned myself. I keep reminding to myself what the staff told me about how to be confident. I go to the gym, I participate in different teams, I have developed interests in my life. Unusual feelings of love for life*…”

The role of exercise in the empowerment of chronic patients is also reported by Leamy et al. (14), thus indicating the embodied aspect of recovery, which is not confined only to mental activities and changes, but actively involves in the process the whole body of the individual.

Empowerment also includes the ability of self-care and self-organization, as described by H.T.:

“*I now have a better understanding of the value of money, not to waste and to do planning … I see the reality … I am organizing better in cleaning, cooking, I can manage better and more easily my transactions with the social services. I can now largely control my mouth, my anger. I have more patience and responsibility. I have set limits to myself.*”

**Figure 2:**
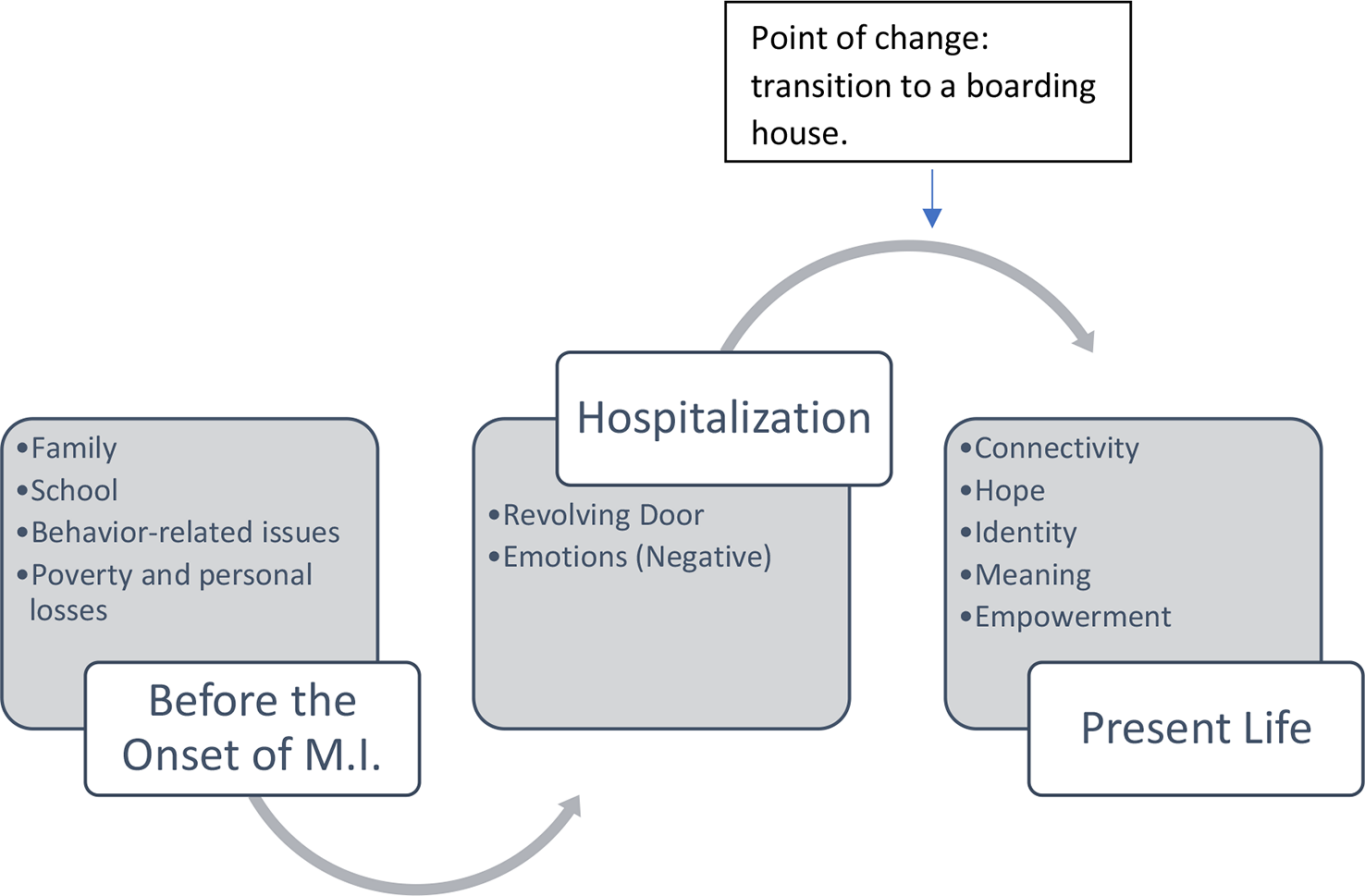
Schematic presentation of the research findings.

In **Figure 2**, a schematic presentation of the research findings is presented.

## Discussion—Implications of the Study

The present study explored the experiences of service users living in EPAPSY’s community residential facilities, taking into account a personal understanding of the recovery and the CHIME conceptual framework. Research findings revealed service users’ lived experiences before, during, and after hospitalization, as they described their early years at home and at school, later on the onset of mental illness and their drifting through the psychiatric hospitals, and finally their transition to a community residential facility and their lives at present. With regard to the CHIME model, findings of the study confirm the five key notions of the model as important aspects of the recovery journey of people with mental health challenges. Specifically, it addresses the subjective aspect of the lived experience of each service user, which lies at the core of every recovery framework (51). Participants underline the sense of freedom they now feel and the personal control they have regained over their everyday life and decisions, emphasizing a sense of positivity and optimism for the future, standing in stark contrast to their bleak and difficult past. Their accounts revealed a developing self-understanding in relation to their biographical experiences, an expression of gratitude for their present life, an appreciation for the present quality of life, and a sense of meaning making and well-being. Their narratives also expressed hopes and dreams for the future and portrayed a personal understanding of mental illness and recovery, a growing sense of autonomy and ability for self-care and self-management skills. Moreover, there was recognition of the importance of relationships and social support. Additionally, as seen in **Figure 2**, the accounts seem to change abruptly from one point on; before their move into a housing facility, they remember a life of negative feelings, multiple losses, sadness, and drifting in and out of psychiatric hospitals. After their move to the housing facility, their accounts change to a positive attitude of hope. Although themes such as childhood and school years may at first seem irrelevant to the CHIME model, still they are especially valuable in that they inform participants’ life and the meaning they attribute to their personal experience, as well as our contextualized understanding of their lifelong journey before and after the onset of mental health challenges.

Given that the change in participants’ accounts begins after their move in a boarding house, it seems likely that the provided services had an impact on their recovery. Described as an “in-between place” (51), services and staff training in boarding houses should therefore be carefully studied and organized in order to ensure an effective and sustaining start in the recovery path of people with mental health challenges. Moreover, participants stress the importance of employment and of independent living as major motives and goals for their life, as well as relationships and an identity of “normality” as key factors of their present life. To that end, available services should be well-trained and properly equipped to provide for occupational education, training, and employment, investing in and refining strengths and available knowledge to the benefit of service users. Given the community perspective and the ongoing stigmatization of mental illness, available residential facilities should also work toward minimizing discriminatory attitudes and behaviors in the local population and the service users’ families through informative programs and open communication channels.

Onken et al. (52) have commented on the positivity associated with recovery-oriented practice and caution that there is a risk that the recovery process may be considered simple and easy for everyone involved. In our study, a positive outlook seemed to coexist with only a slight reservation and with an awareness from the part of the participants that there are obstacles and constraints (e.g., the presence of mental illness and symptoms, reduced ability for a full and complete autonomy and/or independence, work-related obstacles) that could make difficult or postpone the fulfillment of their hopes and dreams. This optimistic view runs the risk of being misinterpreted both by service users and researchers alike. Recovery is not an easy process, and results are never uniform or positive for all users. Moreover, providing recovery services which highlight only the positive aspects of life fails to acknowledge the hardships and the crises that may inflict the lives of all people, at any time, thus remaining overprotective of service users and unrealistic in regard to the expected results. It remains unclear if, in the present study, this uniformity of accounts on the positiveness reported by participants is due to the fact that they were individuals trying to please their therapists, to the participants’ sense of gratitude toward the residential facility, or in fact to a more pragmatic and realistic sense of personal progress and well-being on behalf of participants. Given the negative picture the participants draw on their early years and the period of their hospitalizations, it is possible that their transition to a community residential facility and from there on to a more independent but still protected and more “normal” environment provides them and their accounts with an overwhelming optimism. Nevertheless, the positive outcomes should not lead us to believe that recovery can take place even without the intervention of quality mental health services provided by competent professionals.

In addition, research results indicated that there was some overlap among the notions associated with the five key concepts proposed by the CHIME recovery framework indicating an interconnectedness and complementarity in the context of the recovery process.

Since all recovery processes start with the professional and nonprofessional staff, emphasis should be given in the attitudes of the personnel toward mental illness and the training required to accommodate the needs of the service users. Le Boutillier et al. (53) found that there is no clarity over the theoretical aspects of recovery services provision among personnel, making it hard to discern between the needs of the service users, the needs of the health system, and the needs of the organization. This confusion inhibits the flow of the recovery process and obscures the priorities of the service users. The present study offers a multitude of insights regarding the needs of mental health services users, which can be used in order to inform professional and nonprofessional staff of their core priorities and wishes.

Taking into account the possible benefits for recovery-oriented practice, this seems to be a very promising approach, worthy of further inquiry. Research into the process and meaning of personal recovery is especially important for the continuing development of clinical mental health and recovery-oriented mental health community services. Further qualitative research on the subject in other mental health community residential facilities would also support transferability of the present findings.

### Limitations

The present study is the first in Greece to attempt to inquire into the CHIME recovery model and the lived experiences of people with mental health challenges who live in EPAPSY’s residential facilities. As such, it contributes to our knowledge of the community work being done in the country, having nevertheless certain limitations which provide ample questions for further investigation.

On the methodology applied, the use of participants’ therapists as interviewers may have risked the collection of accounts biased toward a positive extreme. Nevertheless, it was considered the best available option in order not to disrupt participants’ lives and relationships. Given that other qualitative methods, such as participatory observation or using experienced therapists as interviewers, could have been used, it remains open to further qualitative studies to inquire into similar research questions in order to enrich our knowledge of the experience of people facing mental health challenges of the recovery processes in Greece.

Furthermore, the study did not take into account other variables of the recovery process, such as the quality of relationships with the clinical staff, clinical aspects of the recovery, as well as personal elements, such as the motivation to change or their level of cognitive abilities.

Reflecting upon our sample, we consider that it could be larger. Nonetheless, qualitative methods focus on an in-depth understanding of the personal experience, and they do not aim to generalize results; instead, their aim is to inform our understanding of the phenomenon under study and to offer new questions for more focused research (54). Still it should be noted that the small number of participants is due to the fact that researchers asked for participation of only high-functioning service users, able to narrate their life story and to reflect on their experience. If we had the possibility to recruit more participants, richer and more varied accounts would have been reported. Furthermore, the sample is skewed gender-wise due to the fact that sampling was convenient and the individuals who consented to participate were mostly men. Accordingly, due to the small sample, we were not able to achieve data saturation.

## Data Availability Statement

The datasets generated for this study are available on request to the corresponding authors.

## Ethics Statement

Ethical review and approval was not required for the study on human participants in accordance with the local legislation and institutional requirements. The patients/participants provided their written informed consent to participate in this study. Written informed consent was obtained from the individual(s) for the publication of any potentially identifiable images or data included in this article.

## Author Contributions

AAp conducted the thematic analysis, the writing of the findings, and the discussion and coauthored all sections of the article. SS did a critical revision of the article. PI oversaw the methodology of the research and coauthored the methodology, research findings, and discussion sections of the article. PC had the initial conception of the work and coauthored the introduction and literature review sections. AAl, PB, CG, EK, VF, and FT conducted collection of data. 

## Conflict of Interest

The authors declare that the research was conducted in the absence of any commercial or financial relationships that could be construed as a potential conflict of interest.
